# Effect of low-dose Levamlodipine Besylate in the treatment of vascular dementia

**DOI:** 10.1038/s41598-019-47868-0

**Published:** 2019-12-03

**Authors:** Kai-Xin Yao, Hang Lyu, Mei-Hua Liao, Lin Yang, Yin-Ping Gao, Qi-Bing Liu, Cheng-kun Wang, Ying-Mei Lu, Guo-Jun Jiang, Feng Han, Ping Wang

**Affiliations:** 1 College of Pharmaceutical Sciences, Zhejiang University of Technology, Hangzhou, China; 2 College of Pharmaceutical Sciences, Zhejiang University, Hangzhou, Zhejiang China; 3 School of Medicine, Zhejiang University City College, Hangzhou, Zhejiang China; 4 Department of Pharmacy, Zhejiang Xiaoshan Hospital, Hangzhou, Zhejiang China; 5 School of Pharmacy, Hainan Medical College, Haikou, China

**Keywords:** Mechanisms of disease, Mechanisms of disease, Mechanisms of disease, Mechanisms of disease, Mechanisms of disease

## Abstract

Vascular dementia (VaD) is a complex disorder caused by reduced blood flow in the brain. However, there is no effective pharmacological treatment option available until now. Here, we reported that low-dose levamlodipine besylate could reverse the cognitive impairment in VaD mice model of right unilateral common carotid arteries occlusion (rUCCAO). Oral administration of levamlodipine besylate (0.1 mg/kg) could reduce the latency to find the hidden platform in the MWM test as compared to the vehicle group. Furthermore, vehicle-treated mice revealed reduced phospho-CaMKII (Thr286) levels in the hippocampus, which can be partially restored by levamlodipine besylate (0.1 mg/kg and 0.5 mg/kg) treatment. No significant outcome on microglia and astrocytes were observed following levamlodipine besylate treatment. This data reveal novel findings of the therapeutic potential of low-dose levamlodipine besylate that could considerably enhance the cognitive function in VaD mice.

## Introduction

Vascular dementia (VaD), which is usually caused by cerebrovascular disease, often attributes to cognitive decline^1–3^. Considerable research concerning the molecular biological basis of VaD has been done, but unfortunately, few of them have yet to be translated into a new medication for this disease^4–6^. Therefore, the development of more effective drugs for the treatment of VaD is of great importance.

Disturbance of the intracellular calcium homeostasis is central to the pathophysiology of neurodegeneration^7–9^. Vascular dementia is caused by cerebral hypoperfusion and may benefit from calcium channel blockade due to the relaxation of the cerebral vasculature^10,11^. Indeed, calcium channel blockers (CCB) may be an exciting class of therapies as they may improve cerebrovascular perfusion^12^. In addition, the calmodulin inhibitor alleviates the hippocampus-dependent spatial cognition dysfunction in a murine model of VaD^13^. Several CCBs have been tested in clinical trials of dementia, and the result is heterogeneous. Nilvadipine, as well as nimodipine, prevents cognitive decline in some trials, whereas other CCBs failed^14,15^. Therefore, due to the absence of ideal antidementia drugs, a testing novel CCBs with improved physicochemical properties may be valuable.

Levamlodipine, also known as S-amlodipine or levoamlodipine, is a pharmacologically active enantiomer of amlodipine. Receptor binding studies have shown that contrary to the (R)-enantiomer, (S)-enantiomer can bind to and block L-type calcium channels^16^. Recent research shows that levamlodipine can prevent the transmembrane influx of calcium into the vascular and cardiac smooth muscle cells, which resulted in vasodilatation and hence a fall in blood pressure^17^. Therefore, levamlodipine can be a prospective pharmacological treatment for VaD. Here, we demonstrated that low-dose levamlodipine treatment could mitigate the hippocampus-dependent spatial cognition disorder in a murine model of VaD. In addition, this pharmacological outcome was associated with a marked restoration of hippocampal Ca^2+^/CaM-dependent protein kinase II (CaMKII) in mice after eight weeks-right unilateral common carotid arteries occlusion (rUCCAO).

## Results

### Levamlodipine Besylate restores spatial learning and memory in VaD mice

**Figure 1:**
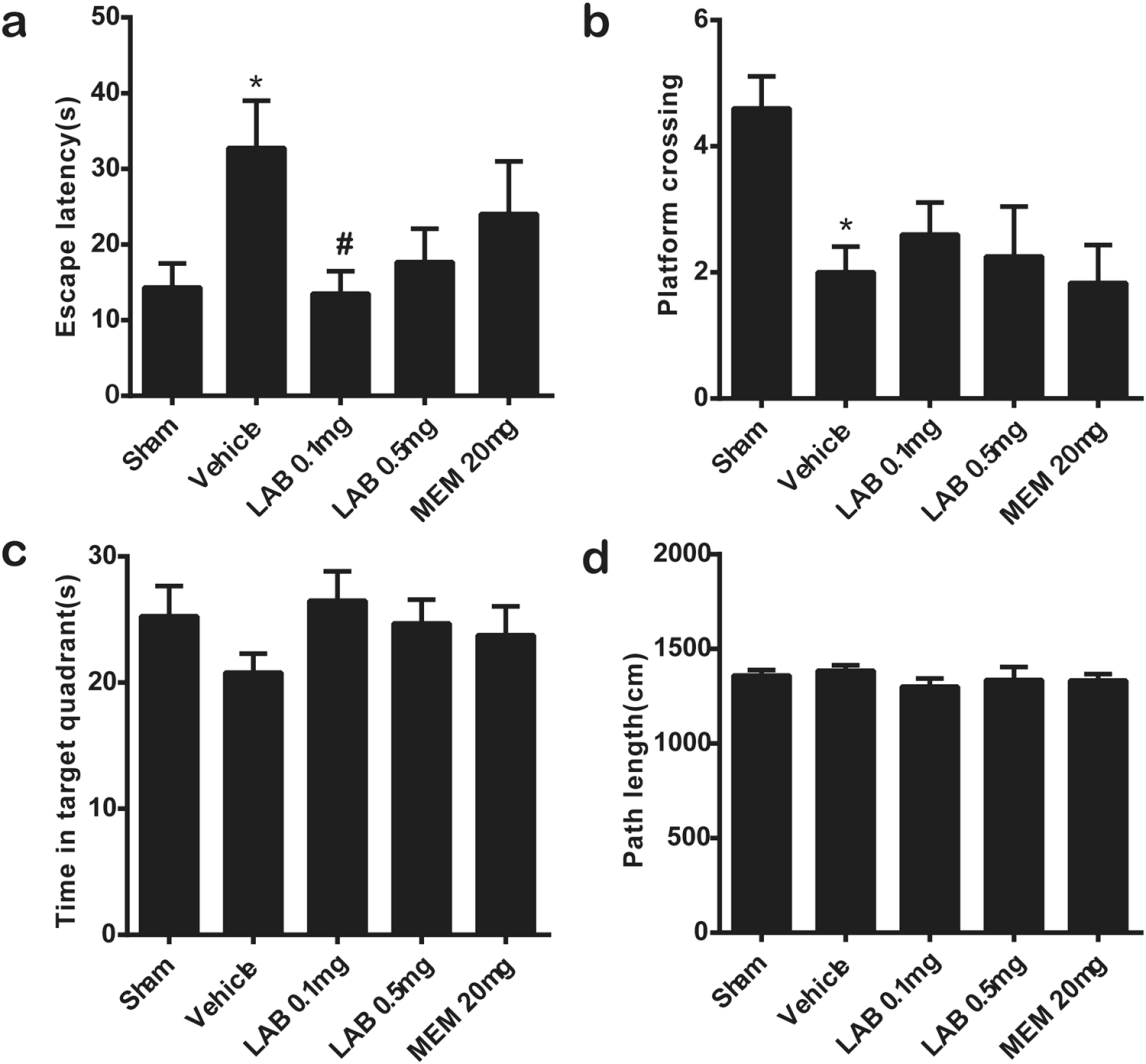
Levamlodipine Besylate restores spatial learning and memory in VaD mice. Eight weeks after rUCCAO operation, Levamlodipine Besylate was administered orally (*p.o*.) at 0.1 or 0.5 mg/kg, and Mementine was administered orally (*p.o*.) at a dose of 20 mg/kg for eight weeks. The Morris water maze experiment was used to test the spatial learning of these mice. (**a**) The changes in escape latency were probed to find the hidden platform produced during training trials in each group. *f_(10,10)_ = 5.97, ^#^*f*_(10,10)_ = 3.44, *p < 0.05 *verse* sham group; ^#^*p* < 0.05 *verse* vehicle group. (means ± SEM, n = 10 mice in each group) (**b**). On the fifth day, each mouse was tested in a probe trial by removing the platform from the pool. The platform crossing time were recorded. *f_(10,10)_ = 4.43, *p < 0.05 *verse* sham grou*p*. (means ± SEM, n = 10 mice in each group) (**c**). Time spent in target quadrant in the test day (means ± SEM, n = 10 mice in each group) (**d**). Path length of mice during the MWM test. (means ± SEM, n = 10 mice in each group).

In the present study, we investigated the pharmacological outcome of low-dose levamlodipine besylate on hypoperfusion-induced VaD. Levamlodipine was often used at the dose of 0.05 mg/kg on patients in clinical research^18^. According to the guide for dose conversion between animals and human, relevant papers choose the dosage of 1 mg/kg in rats^19^. Considering the eight-week chronic administration for mice, we select the dose of 0.1 mg/kg and 0.5 mg/kg in this study. Compared with sham-operated mice, the escape latency to find the hidden platform during training trials was significantly impaired in the vehicle-treated rUCCAO mice (*P* < 0.05). Notably, levamlodipine besylate’s attenuation to this cognitive dysfunction was observed in mice after daily repeating administration of levamlodipine at the doses of 0.1 mg/kg (Fig. 1).

**Figure 2:**
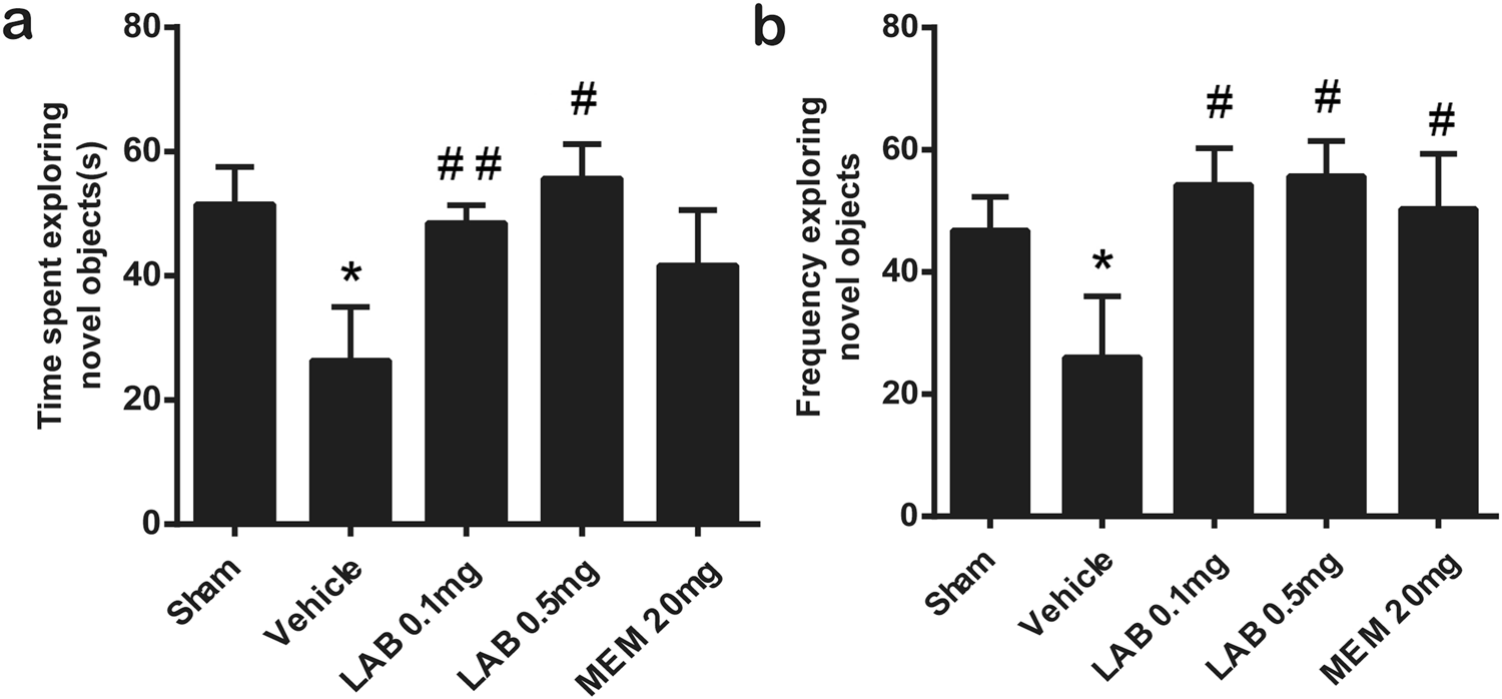
Levamlodipine Besylate can improve the recognition memory impaired in VaD mice. The novel object recognition test (NORT) was used to evaluate recognition memory. The time spent exploring novel object (**a**) and frequency exploring novel object (**b**) were recorded and analyze. *p < 0.05 *verse* sham group; ^#^*p* < 0.05 *verse* vehicle group; ^##^*p* < 0.01 *verse* vehicle group. (means ± SEM, n = 12 mice in each group).

In the novel object recognition (NOR) task, vehicle-treated rUCCAO mice showed a reduced ability to discriminate familiar object from novel one compared with sham mice (Fig. 2). By contrast, for the levamlodipine besylate (0.1 or 0.5 mg/kg)-treated rUCCAO mice, a significant improvement of cognitive function was observed, as revealed by significantly elevated exploring time (Fig. 2a) and exploring frequency (Fig. 2b) on different objects as compared to vehicle-treated mice.

### Levamlodipine besylate prevents the dephosphorylation of CaMKII in rUCCAO mice

**Figure 3:**
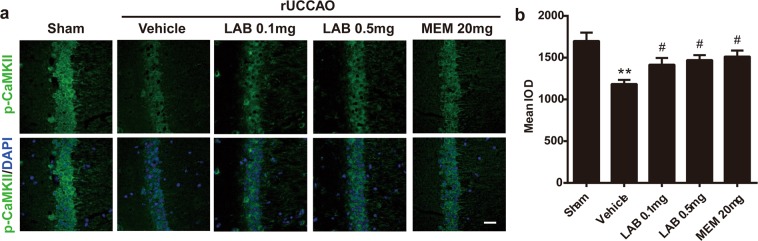
Levamlodipine Besylate prevents the dephosphorylation of CaMKII in rUCCAO mice. A significant decrease in the intensity of fluorescence for phospho-CaMKII (Thr286) in CA1 pyramidal neurons of the hippocampus. Levamlodipine Besylate (0.1 mg/kg) could restore the decrease in immunostaining for phospho-CaMKII (Thr286). (**a**) Representative images of phospho-CaMKII in hippocampus CA1 region. (**b**) Quantification of phospho-CaMKII in CA1 region.

We, therefore, investigated some representative biochemical events to support the behavioral observations above. CaMKII is localized subcellular to the dendrites and the postsynaptic densities of excitatory synapses, and its phosphorylation was measured as a significant mediator of learning and memory^20^. Here, immunofluorescence staining was performed to further confirm the outcome of levamlodipine besylate on phospho-CaMKII (Thr286) expression in the hippocampal region in rUCCAO mice. As revealed in Fig. 3a, a major decrease in the intensity of fluorescence for phospho-CaMKII (Thr286) in cornu ammonis 1 (CA1) pyramidal neurons of the hippocampus in vehicle mice compared with the sham-operated group. By contrast, levamlodipine besylate (0.1 mg/kg and 0.5 mg/kg) restored this decrease (Fig. 3a,b). In addition, memantine (20 mg/kg) could also restore the decrease, indicating that memantine might improve the cognitive dysfunction in VaD mice.

### Effect of levamlodipine besylate on blood vessels in rUCCAO mice

**Figure 4:**
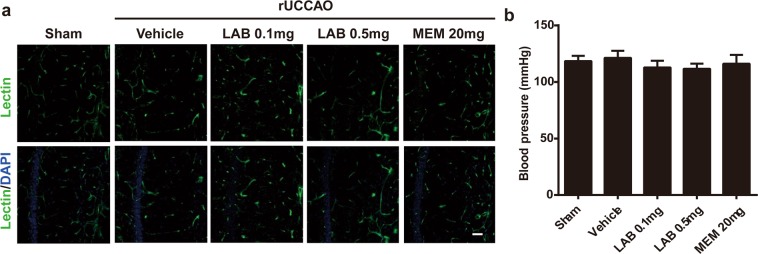
Effect of Levamlodipine Besylate on brain vascular structure in rUCCAO mice. (**a**) Representative immunochemistry image of brain blood vessels in hippocampus CA1 region. No significant effect on the vascular structure were observed following Levamlodipine Besylate (0.1 mg/kg and 0.5 mg/kg) or mementine (20 mg/kg) treatment. (**b**) Blood pressure of different group of mice.

Brain vascular deficit contributes to the progress of VaD^21^. Here, we observed no obvious changes in vascular structure between sham and vehicle group (Fig. 4a). Levamlodipine besylate (0.1 mg/kg and 0.5 mg/kg) or memantine (20 mg/kg) treatment also did not have effect on their structure (Fig. 4a). There were also no differences in blood pressure among all groups (Fig. 4b).

### Effect of levamlodipine Besylate on astrocyte activation in rUCCAO mice

**Figure 5:**
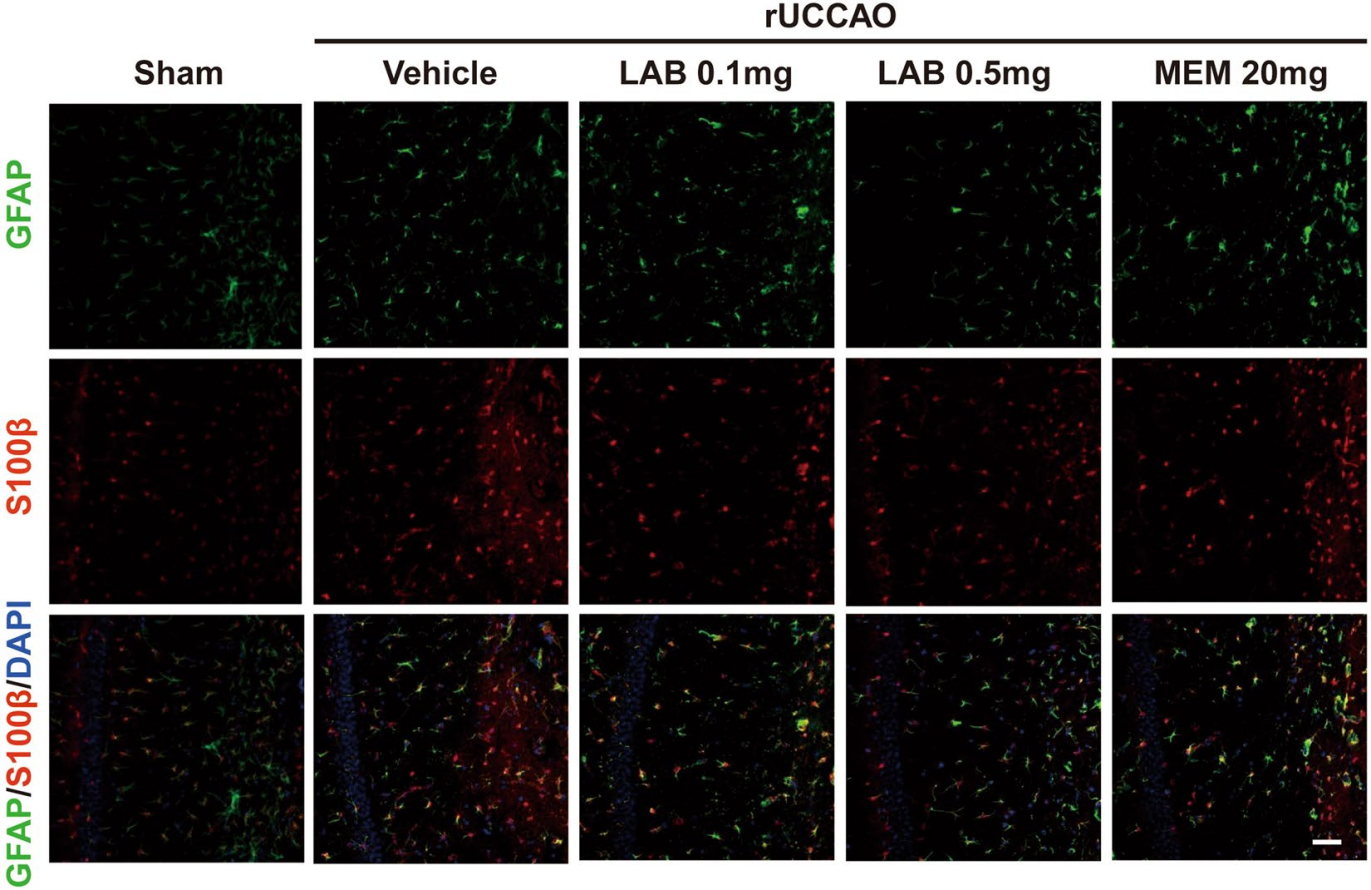
Effect of Levamlodipine Besylate on the activation of astrocyte in rUCCAO mice. Levamlodipine Besylate (0.1 mg/kg and 0.5 mg/kg) or mementine (20 mg/kg) treatment has no significant effect on the activation of astrocyte.

Accumulating evidence showed that astrocytes were activated during the pathological process of VaD^22^. Here, we observe a dramatic activation of astrocytes, as indicated by the elevation of GFAP expression. The data demonstrated that there was no significant inhibitory effect on astrocytes activation following levamlodipine besylate (0.1 mg/kg and 0.5 mg/kg) or memantine (20 mg/kg) treatment (Fig. 5). To extend our observations on astrocytes activation, we tested the total number of astrocytes by using S100β, an astrocytes marker. A similar result was also observed in CA1 regions of the hippocampus (Fig. 5).

### Effect of levamlodipine besylate on microglia in rUCCAO mice

**Figure 6:**
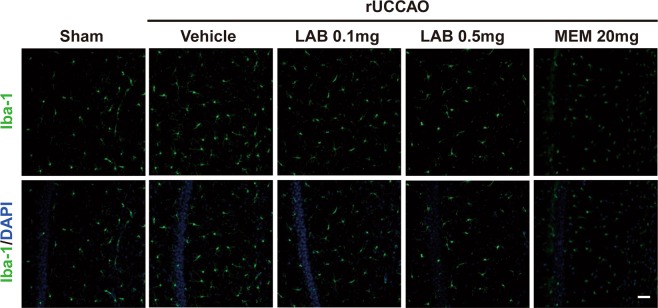
Effect of Levamlodipine Besylate on microglia activation in rUCCAO mice. Iba-1 expressed in the hippocampus neurons of vehicle group was significantly increased in rUCCAO mice. Levamlodipine Besylate (0.1 mg/kg and 0.5 mg/kg) or mementine (20 mg/kg) treatment did not significantly attenuated rUCCAO-induced microglia activation in the hippocampus.

Microglia-induced neurotoxicity may contribute to the development of neurodegeneration in response to pathological signals by stimulating morphological changes and the production of a wide array of inflammatory cytokines^23^. We further explore the effect of levamlodipine besylate on microglia in rUCCAO mice. Unlike a sham-operated group, our data revealed that the number of Iba-1 expressed cells in the hippocampus CA1 region of vehicle group was considerably increased. Here, levamlodipine besylate (0.1 mg/kg and 0.5 mg/kg) or memantine (20 mg/kg) treatment did not significantly attenuated rUCCAO-induced microglia activation in the hippocampus (Fig. 6).

## Discussion

Brain hypoperfusion consequently results in a defect of learning and memory^24^. In the present study, we revealed that hypoperfusion mediated dephosphorylation of CaMKII contributes to cognitive dysfunction, which can be rescued by levamlodipine besylate treatment. Overall, our findings add further support to the anti-dementia effect of levamlodipine besylate observed during behavioral and biochemical studies.

Importantly, low-dose levamlodipine besylate results in reduced neurological dysfunction after VaD. Several studies suggest that low-dose memantine could block the mild N-methyl-D-aspartate (NMDA) receptor and therefore improved working memory related to a highly challenging task in naïve rats^25,26^. However, the use of high dose NMDA receptor antagonists, such as memantine and MK-801, could impair the cognitive function^27^. Therefore, the low-dose levamlodipine besylate might be suitable for VaD treatment, might be due to less effect on the blood pressure. Blood pressure reduction seems not to play a role in preventing dementia, indicating a direct protecting impact on neurons. Optimization of CCBs for the management of dementia may involve enhancement of the affinity and an increase of selectivity for presynaptic calcium channels to the inactivated state.

CaMKII is widely disseminated in the brain and a key mediator of physiological excitatory glutamate signals underlying long-term potentiation (LTP) induction and maintenance^28–31^. Compared with behavior task, we suggested that dephosphorylation of CaMKII at Thr286 is a more sensitive indicator of learning and memory impairment. In the present study, we found that levamlodipine besylate (at the dose of 0.1 mg/kg and 0.5 mg/kg) could reverse the auto-phosphorylation of CaMKII at threonine 286 in CA1 regions of the hippocampus. The activation of CaMKII signaling contributes to the development of hypoperfusion-induced memory and learning deficits^32,33^. A surplus of intracellular calcium is harmful to neuronal function^34^. Therefore, these results suggest that low-dose levamlodipine besylate could considerably improve the cognitive dysfunction in VaD mice. We recently reported that calmodulin inhibitor somewhat introverted the dephosphorylation of CaMKII and synapsin I and amplified the number of mature neurons in the hippocampus, which associated with a major improvement in cognitive dysfunction in VaD mice^12^.

Whereas the present study has provided helpful information about the effect of low-dose levamlodipine besylate in the management of VaD, but it has several limitations that must be acknowledged. This study offered little details on toxicity pieces of evidence of high dosage levamlodipine besylate in the present context. We were unable to conduct various trails with a different length of time for levamlodipine besylate treatment. This suggests the need for a multi-faceted and combination design, perhaps including more extensive time frame and animal models in dementia settings. The design of more potent and selective CCBs with a high level of state dependency may open avenues for new antidementive medication.

## Materials and Methods

### Chemicals

All chemical reagents were obtained from Sigma-Aldrich (St. Lou with an average molecular is, MO, USA) except as otherwise stated. Levamlodipine Besylate was supplied by Shi huida Pharmaceutical Group (Shanghai).

### Ethics statement

All experimental procedures and animal handling protocols were conducted according to the National Institutes of Health (NIH) guidelines for the care and use of laboratory animals. These procedures were approved by the Committee for Ethics of Animal Experiments at the Zhejiang University, China.

### Animal model of rUCCAO

The rUCCAO model was prepared as previously reported^35^. Animals were divided into five groups. First, mice were anesthetized with chloral hydrate (400 mg/kg) and through midline cervical incision, the right common carotid artery (CCA) was isolated from the adjacent vagus nerve and double-ligated with 6–0 silk sutures. Sham-operated mice were subjected to the same surgical procedure without carotid ligation. After the operation, the mice were kept in their quarters with food and water available ad libitum. Eight weeks after operation, Levamlodipine Besylate was administered orally (*p.o*.) at 0.1 or 0.5 mg/kg respectively to the group of LAB 0.1 mg or LAB 0.5 mg, and Mementine was administered orally (*p.o*.) at a dose of 20 mg/kg to the group of MEM 20 mg. Sham and Vehicle group were administered saline orally. All the drugs were dissolved in saline. Drugs were administering once a day for eight weeks.

### Behavioral tests

The novel object recognition test (NORT) was used to evaluate recognition memory^20^. Briefly, mice were individually habituated to an open field box (35 × 25 × 35 cm) for two consecutive days. The experimenter who scored the behavior was blind to the treatment. Discrimination index was evaluated by comparing the difference between the time of exploration of the novel and familiar object and the total time spent on exploring both objects, which made it possible to adjust for differences in total exploration time.

Acquisition of the spatial learning task was performed over 4 consecutive days of testing as previously reported using the Morris water maze Task^36^. The mouse was guided to the platform by the experimenter. The mouse spent another 10 s on the platform before it was picked up and placed back in the home cage. The day before the first day of formal test, pre-training sessions consisting of four trials (trial intervals of 45–60 min) were performed using a visible platform to exclude visual deficient mice. On the fifth day, each mouse was tested in a probe trial by removing the platform from the pool. The data were processed and analyzed for each acquisition trial or probe trial as the escape latency (in seconds) and the times of platform crossing (in numbers) time in the target quadrant (in seconds).

### Immunohistochemical staining and analysis

After behavior test, animals were intracardially perfused with PBS followed by 4% PFA as previously described^37,38^. Briefly, the brain sections were cut and incubated at room temperature in PBS with 0.01% Triton X-100 for 30 min and followed by blocking with 3% bovine serum albumin for 1 h. For immunolabeling, brain slices were probed with primary antibody overnight at 4 °C. Antibodies included phospho-CaMKII (Thr286) (1:200)^39,40^, laminin, GFAP, OX-42, and S100β (1:500, Millipore, U.S.A), Nuclei were stained with DAPI (4, 6-diamidino-2- phenylindole) (Sigma-Aldrich, U.S.A). After washing, the sections were incubated with Alexa Fluor 488-conjugated anti-rabbit IgG and Alexa Fluor 594-conjugated anti-mouse IgG (Invitrogen, Carlsbad, CA). Signals were visualized by using a Zeiss LSM 510 confocal microscope. The relative fluorescence intensity of immunostaining was quantified by using Image J software (NIH, Bethesda, MD, USA).

### Statistical analysis

The data are presented as the mean ± S.E.M. Statistical significance was determined using one-way analysis of variance (ANOVA) followed by Tukey’s test for multigroup comparisons. *P* < 0.05 indicated statistically significant differences.
